# Unlocking the hidden burden of epilepsy in Africa: Understanding the challenges and harnessing opportunities for improved care

**DOI:** 10.1002/hsr2.1220

**Published:** 2023-04-17

**Authors:** Aderinto Nicholas

**Affiliations:** ^1^ Department of Medicine and Surgery Ladoke Akintola University of Technology Ogbomoso Nigeria

**Keywords:** Africa, epilepsy, neurology

## Abstract

**Background:**

Epilepsy is a common neurological disorder that affects many people in Africa, yet the burden of the disease is often hidden. The management of epilepsy in Africa is often inadequate due to a lack of awareness and education, limited access to diagnostic tools and treatments, inadequate coordination of care, and the stigma associated with the condition.

**Method:**

A comprehensive literature review was conducted to gather information on the epidemiology, diagnosis, management, and outcomes of epilepsy in Africa. The review included studies from peer‐reviewed journals, government reports, and gray literature.

**Results:**

The review revealed a high burden of epilepsy in Africa. The studies also showed a significant gap in the availability and accessibility of diagnostic tools, antiepileptic drugs, and specialized therapies such as physical, occupational, and speech therapy. Additionally, the review found that cultural beliefs and practices, socioeconomic factors such as poverty and lack of access to healthcare, and the stigma associated with the condition, also pose significant challenges to managing epilepsy in Africa.

**Conclusion:**

The study highlights the urgent need for improved epilepsy management in Africa. Additionally, the study calls for further research on epilepsy in Africa and collaboration between all stakeholders to improve the management of epilepsy in Africa.

## 1 INTRODUCTION

Epilepsy is a neurological disorder characterized by recurrent seizures, affecting more than 50 million individuals globally, with a significant number residing in Africa.^1^, ^2^ The World Health Organization (WHO) estimates that approximately 25 million people in Africa have epilepsy, and 80% live in low‐ and middle‐income countries.^3^ Unfortunately, despite the considerable burden of this disease in Africa, patient care and support are inadequate.^4^

The challenges associated with epilepsy management in Africa include limited access to diagnostic tools and treatments, inadequate care coordination, and a lack of disease awareness and understanding.^5^, ^6^, ^7^ Given that Africa is home to culturally and socioeconomically diverse populations, these unique challenges must be addressed when developing interventions for epilepsy care. In addition, studying epilepsy in Africa can provide valuable insights into managing the disease, particularly regarding cultural and socioeconomic factors that differ from other regions. Such insights can help develop culturally appropriate and effective interventions to enhance the quality of life of people with epilepsy in Africa.

This paper examines epilepsy management in Africa, encompassing challenges and opportunities for improvement. The implications of this paper are substantial for healthcare professionals, policymakers, and stakeholders in epilepsy care. The aim is to provide insights for effective interventions to enhance patients’ quality of life in Africa.

## 2 EPIDEMIOLOGY OF EPILEPSY IN AFRICA

Epilepsy is a significant public health concern in Africa, with a high prevalence and incidence rate. The epidemiology of epilepsy in Africa varies widely across different countries and populations studied.^8^, ^9^ Several studies have reported a prevalence of epilepsy ranging from 0.5% to 10% in various African countries.^10^, ^11^, ^12^, ^13^ However, it is widely believed that these figures may be underestimated due to underdiagnosis and underreporting of the disease. For instance, Ghana has an estimated prevalence of 7.9%, while Tanzania has a prevalence as high as 10%.^12^ In terms of incidence, studies have reported rates ranging from 20 to 50 per 100,000 people per year in different African countries.^14^ For example, Ghana has an estimated incidence of 40 per 100,000 people per year, while Ethiopia has an estimated incidence of 30 per 100,000 people per year.^14^, ^15^ Nigeria, South Africa, and other countries have estimated incidence rates of 27 and 25 per 100,000 people per year, respectively.^16^, ^17^, ^18^

A striking finding is the prevalence and incidence of epilepsy in rural areas compared to urban areas.^18^ This disparity is likely due to the lack of access to healthcare and diagnostic tools in rural areas and the limited awareness of the signs and symptoms of epilepsy, making it more difficult to diagnose and treat the condition. Moreover, a higher prevalence and incidence of epilepsy are observed in low‐ and middle‐income countries, which lack resources and healthcare infrastructure, leading to difficulties in diagnosis and treatment.^19^ Similarly, the burden of epilepsy is higher among children and young adults in Africa, likely due to a higher incidence of infectious diseases and traumatic injuries, known risk factors for epilepsy.^20^ Notably, studies have revealed a higher prevalence and incidence of idiopathic epilepsy (epilepsy with no known cause) in Africa compared to symptomatic epilepsy (epilepsy caused by an underlying condition).^21^ This finding underscores the importance of continued research efforts to understand the unique factors contributing to the high incidence and prevalence of idiopathic epilepsy in Africa.

## 3 CULTURAL, SOCIOECONOMIC, AND HEALTHCARE FACTORS AFFECTING EPILEPSY MANAGEMENT IN AFRICA

Cultural beliefs and practices are essential to managing epilepsy in Africa. However, they can have positive and negative impacts on managing the disease, highlighting the need for a nuanced understanding of their role. Traditional healing practices and herbal medicines can support people with epilepsy in Africa, providing emotional support and access to affordable and accessible treatments. In Ghana, for example, traditional healers play an essential role in managing epilepsy, with many people consulting them in addition to or instead of conventional healthcare providers.^22^ This underscores the importance of incorporating traditional healing practices into the healthcare system and improving collaboration between traditional healers and conventional healthcare providers. Despite these benefits, cultural beliefs and practices can also negatively impact management. Cultural beliefs may lead to stigmatization and discrimination against people with epilepsy, affecting their quality of life. Moreover, some traditional practices, such as restrictive and dangerous restraint methods, can be harmful and even life‐threatening to people with epilepsy.^23^ Therefore, it is critical to raise awareness about the negative impacts of cultural beliefs and practices and promote the adoption of evidence‐based management strategies to ensure safe and effective treatment for people living with epilepsy in Africa.

Socioeconomic factors such as poverty and lack of access to healthcare are significant challenges for people with epilepsy in Africa.^24^ Poverty leads to difficulty accessing diagnostic tools and treatments for epilepsy, and people living in poverty cannot afford to travel to healthcare facilities or take time off work to seek medical care. In addition, the shortage of trained healthcare professionals such as neurologists and epileptologists, coupled with an overburdened healthcare system and poor quality of care, makes it difficult for people with epilepsy to receive appropriate care and treatment.^24^ Addressing these cultural beliefs and practices and socioeconomic factors is critical in improving the care and management of epilepsy in Africa. This can be achieved by increasing access to healthcare facilities and diagnostic tools, improving the quality of care, providing education and awareness programs to reduce stigma and discrimination, and involving traditional healers in managing epilepsy.

**Figure 1:**
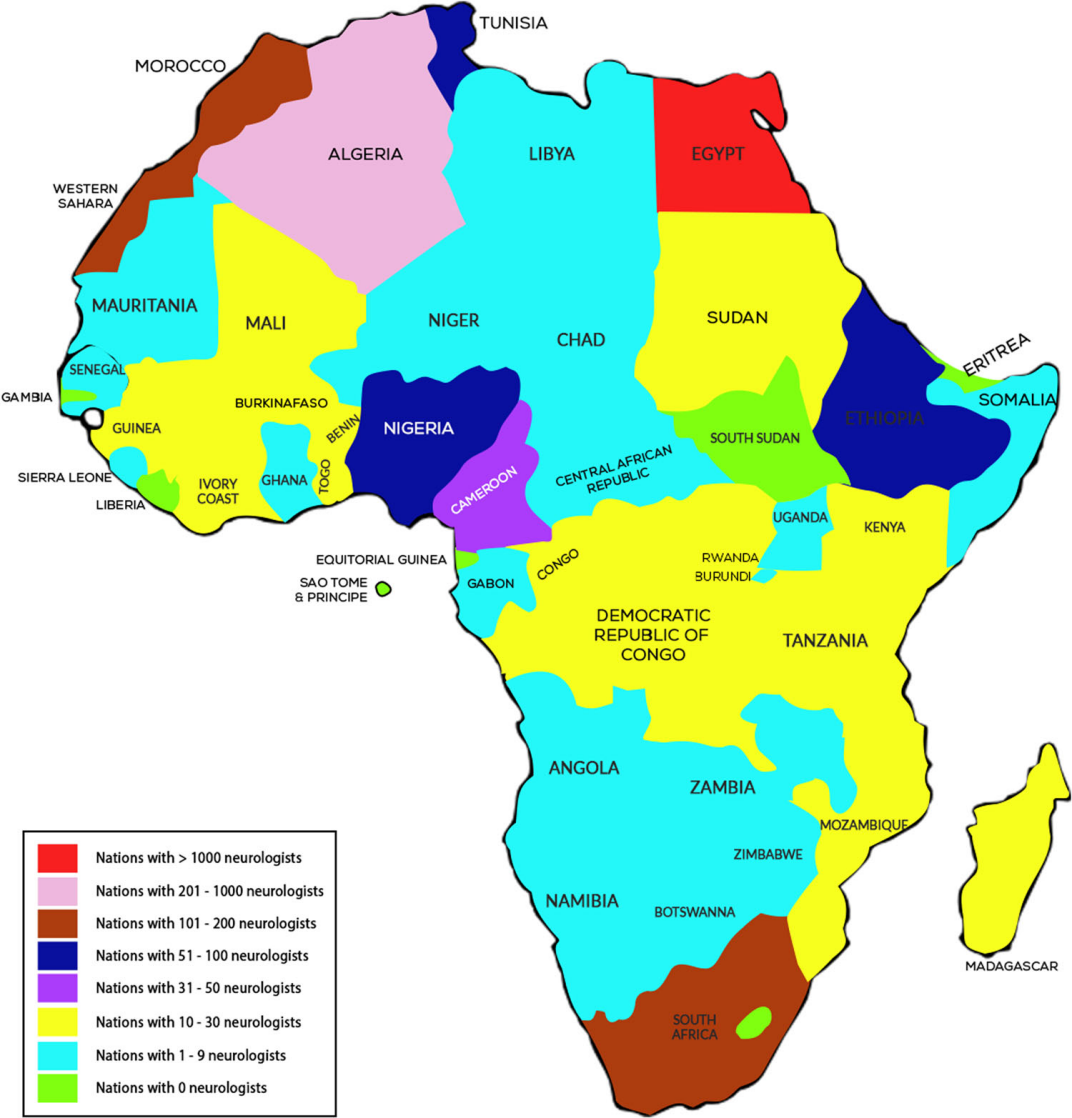
Map showing the number of available neurologists in each African country.^24^

The need for more availability of diagnostic tools in rural and remote areas poses a significant obstacle to a proper diagnosis. With a scarcity of trained healthcare professionals, such as neurologists and epileptologists, to conduct diagnostic tests, the situation is further compounded. Figure 1 shows the number of practicing neurologists in each African country. This lack of access to appropriate diagnostic services can lead to inaccurate or delayed diagnoses, resulting in suboptimal health outcomes for those with epilepsy.

The high cost of diagnostic tools and treatments is also a major challenge, with many African people unable to afford the necessary expenses. Table 1 shows the estimated treatment cost of epilepsy in Africa. Moreover, the availability and affordability of antiepileptic drugs (AEDs) are limited in many African countries.^25^ Without access to the necessary drugs, individuals with epilepsy are at risk of continued seizures, leading to adverse health outcomes.

**Table 1:** Estimated cost of epilepsy treatment in africa.^24^

Medication	$5–$50 per month
Investigations	EEG: $50–$200 MRI: $250–$1000
Health insurance	In many African countries, access to health insurance can be limited, particularly for those living in rural or low‐income areas. Health insurance premiums range from $10 to $50 per month in Africa.

## 4 INTERVENTIONS IN IMPROVING EPILEPSY MANAGEMENT IN AFRICA

Health education programs are a crucial aspect of enhancing the management of epilepsy in Africa. These programs can prove effective in enhancing awareness and understanding of the condition while increasing access to diagnostic tools and treatments. A primary advantage of health education programs for epilepsy in Africa is their potential to increase public awareness and understanding of the condition. Such programs can aid in dismantling misconceptions and stigmas associated with epilepsy, leading to improved early diagnosis and treatment of the condition. Additionally, health education programs for epilepsy in Africa can enhance access to diagnostic tools and treatments. By imparting knowledge about the significance of diagnostic tests and the available types of tests, such programs can increase the number of people accessing these tools and treatments, thereby increasing the likelihood of early diagnosis and treatment. Health education programs can also disseminate information regarding the available treatments for epilepsy and increase access to these treatments. This may include information about AEDs and other treatments, such as surgery and rehabilitation. Furthermore, health education programs can cater to specific populations, such as children and women, and provide relevant information and education on epilepsy management. This helps address the unique needs and concerns of these populations.

Support groups for epilepsy in Africa are pivotal in improving the management and care of people with the condition. These groups can provide several benefits, including emotional support, education, and resource access. One significant advantage of support groups for epilepsy in Africa is their potential to offer emotional support to people with the condition. By providing a safe and supportive space for people to share their experiences and feelings and receive validation and understanding from others living with epilepsy, support groups can aid in mitigating the emotional impact of the condition.

In Africa, mobile health clinics (MHCs) have emerged as a promising strategy to improve the management and care of people living with epilepsy.^25^ MHCs can offer a range of benefits, including improving access to diagnostic tools and treatments and care coordination. People living with epilepsy often face multiple barriers to accessing healthcare, such as long distances to clinics, lack of transportation, and financial constraints. MHCs can provide healthcare services directly to people in their communities, reducing the need for individuals to travel long distances to access care. MHCs can also improve care coordination, particularly for people with epilepsy who receive care from multiple healthcare providers, including primary care physicians, neurologists, and traditional healers.

Rehabilitation in the form of physical, occupational, and speech therapy are critical in managing epilepsy in Africa. They can help improve the quality of life of people with the condition. These therapies address the various physical, cognitive, and emotional challenges individuals with epilepsy may encounter.

Physical therapy can enhance muscle strength, coordination, and balance, which seizures can affect.^26^ Occupational therapy can support people living with epilepsy in developing skills to perform daily activities and adjust to their surroundings.^27^ Speech therapy can help individuals with epilepsy to improve communication skills affected by seizures and medication.^28^ Furthermore, these multidisciplinary therapies can also help people living with epilepsy manage the psychological and emotional impact of the condition. These therapies can aid in coping with the emotional and psychological challenges linked to epilepsy, such as anxiety, depression, and stress.

## 5 CONCLUSION

Epilepsy represents a pressing public health concern in Africa. There is an urgent need to tackle the existing challenges and leverage opportunities for enhanced management and care of the condition. To improve the lives of people with epilepsy in Africa, a comprehensive approach is necessary, encompassing a range of strategies such as increasing awareness and education, promoting access to diagnostic tools and treatments, fostering interdisciplinary collaborations, and supporting epilepsy research in Africa. Further research is essentially better to understand the epidemiology and impact of epilepsy in Africa and to develop culturally tailored interventions.

## AUTHOR CONTRIBUTIONS

**Aderinto Nicholas**: Conceptualization; data curation; investigation; methodology; project administration; resources; supervision; validation; writing—original draft; writing—review and editing.

## TRANSPARENCY STATEMENT

The lead author Aderinto Nicholas affirms that this manuscript is an honest, accurate, and transparent account of the study being reported; that no important aspects of the study have been omitted; and that any discrepancies from the study as planned (and, if relevant, registered) have been explained.

## Data Availability

Data sharing not applicable to this article as no datasets were generated or analyzed during the current study.
